# Long‐term fatigue following aneurysmal subarachnoid haemorrhage and the impact on employment

**DOI:** 10.1111/ene.15533

**Published:** 2022-09-12

**Authors:** Ben Gaastra, Harry Carmichael, Ian Galea, Diederik Bulters

**Affiliations:** ^1^ Clinical Neurosciences, Clinical and Experimental Sciences, Faculty of Medicine University of Southampton Southampton UK; ^2^ Department of Neurosurgery, Wessex Neurological Centre University Hospital Southampton NHS Foundation Trust Southampton UK

**Keywords:** employment, fatigue, outcome, subarachnoid haemorrhage

## Abstract

**Background and purpose:**

Fatigue is common following aneurysmal subarachnoid haemorrhage (aSAH) but little is known about its frequency, prognosis and impact on employment. The aim of this study was to assess the frequency of fatigue, whether it changes over time and the relationship to employment in the long term.

**Methods:**

This was a retrospective observational study of aSAH cases and matched controls from the UK Biobank. The presence of fatigue was compared between cases and controls using the chi‐squared test. The change in frequency over time was assessed using Spearman's rank correlation coefficient. The effect of fatigue on employment was assessed using mediation analysis.

**Results:**

Fatigue is more common following aSAH compared to matched controls (aSAH 18.7%; controls 13.7%; *χ* ^2^ = 13.0, *p* < 0.001) at a mean follow‐up of 123 months. Fatigue gradually improves over time with significant fatigue decreasing by 50% from ~20% in the first year to ~10% after a decade (*p* = 0.04). Fatigue significantly mediated 24.0% of the effect of aSAH status on employment.

**Conclusions:**

Fatigue is common following aSAH and persists in the long term. It gradually improves over time but has a major impact on aSAH survivors, significantly contributing to unemployment following haemorrhage. Further work is required to develop treatments and management strategies for fatigue with a view to improving this symptom and consequently employment following aSAH.

## INTRODUCTION

Aneurysmal subarachnoid haemorrhage (aSAH) is a devastating form of stroke associated with significant morbidity and mortality. It affects younger people than other stroke types, resulting in a disproportionately high socio‐economic impact due to loss of productive employment and the long‐term healthcare burden [1]. Survivors of aSAH can suffer a wide range of neurological deficits ranging from physical disability to less obvious, yet life changing, sequelae including cognitive [2], psychological [3] and auditory deficits [4, 5]. These disabilities contribute to unemployment following aSAH, with up to 50% of previously employed individuals not returning to work at 1 year following haemorrhage [6].

Fatigue is another common consequence of aSAH with one analysis reporting a weighted mean fatigue frequency of 73.6% in the first year, falling to 50.7% thereafter, based on five published studies [7]. Of the studies included in that analysis, where the subarachnoid haemorrhage was confirmed to be aneurysmal, the maximum follow‐up time period was 4 years. Very little is known about the long‐term prognosis of fatigue following aSAH.

Fatigue has significant implications for patients and has been associated with reduced quality of life and impaired return to work following aSAH [3, 8, 9]. A number of factors have been reported to predict fatigue following aSAH including smoking, impaired consciousness, hydrocephalus, anxiety and depression [8, 9, 10, 11].

The aim of this study was (i) to assess the frequency and phenotype of fatigue in the long term following aSAH; (ii) to identify whether the frequency of fatigue changes over time; and (iii) to assess whether fatigue mediated any of the effect of aSAH on employment status.

## METHODS

This was a retrospective case–control study using data from the UK Biobank, a major biomedical database [12]. This study includes information on 502,497 participants with informed consent, aged 40–69 at the time of recruitment between 2006 and 2010 (application ID 49305). The study is reported in accordance with the STROBE statement for case‐controlled studies [13] and has both national REC (16/NW/0274) and institutional approval (ERGO 49253).

### Fatigue

Fatigue was assessed in the UK Biobank at assessment centre visits using the question ‘Over the past 2 weeks, how often have you felt tired or had little energy?’ (data field 2080). Individuals were categorized as suffering significant fatigue if they reported tiredness or little energy for more than half the time. A subset of individuals answered questions about fatigue phenotype (Table 1), scored using a 7‐point scale with a score of 1 indicating strong disagreement and 7 strong agreement. Where applicable correction for multiple testing was performed using the Benjamini–Hochberg procedure with a false discovery rate of 5%.

**TABLE 1:** Questions included from the UK Biobank on fatigue phenotype

Data field	Question
120119	Motivation is lower when fatigued
120120	Exercise brings on fatigue
120121	Easily fatigued
120122	Fatigue interferes with physical functioning
120123	Fatigue causes frequent problems
120124	Fatigue prevents sustained physical functioning
120125	Fatigue interferes with carrying out certain duties and responsibilities
120126	Fatigue is amongst the three most disabling symptoms
120127	Fatigue interferes with work, family or social life

### Aneurysmal subarachnoid haemorrhage population

Aneurysmal subarachnoid haemorrhage cases were identified from the UK Biobank using International Classification of Diseases (ICD) 9 (data field 41271), ICD‐10 (data field 41270), self‐reported medical conditions (data field 20002) and primary care data (data field 42040). Individuals were excluded if the subarachnoid haemorrhage was non‐aneurysmal in nature or if there was a trauma code documented within 30 days of diagnosis (see Table S1 for inclusion and exclusion codes). aSAH cases were included in this study if they had data on fatigue subsequent to the diagnosis of aSAH.

### Control population

A single matched control population was identified from the UK Biobank using propensity score matching with a nearest neighbour method and a case:control ratio of 1:4. Individuals were matched according to age at follow‐up, sex, smoking status and presence of anxiety or depression which have been shown to influence fatigue following aSAH [8, 10, 11]. Smoking status was dichotomized into current smoker or not (data field 20116). Anxiety and depression were dichotomized on whether the individual had seen a doctor for nerves, anxiety, tension or depression (data field 2090). Individuals with missing data on fatigue or covariates were excluded from the control pool available for matching.

### Primary analysis

The chi‐square test was used to compare frequency of fatigue between cases and controls. The *t* test was used to compare fatigue phenotype domains. Spearman's rank correlation coefficient was used to assess the relationship between frequency of fatigue and time.

Severity of clinical presentation and complications of aSAH, such as hydrocephalus, have been shown to be predictive of fatigue [9, 10]. Logistic regression was used to explore whether these features were associated with significant fatigue in this dataset. The dependent variable was significant fatigue with the variable of interest as the independent variable in addition to age, sex, smoking status and presence of anxiety/depression. The presence of hydrocephalus was defined using the Office of Population Censuses and Surveys Classification of Interventions and Procedures (version 4) codes (data field 41272). A201 (drainage of ventricle of brain) and A124 (creation of ventriculo‐peritoneal shunt) at the time of or within 1 year of diagnosis were used. The World Federation of Neurological Surgeons (WFNS) grade is a measure of the severity of clinical presentation and the strongest known predictor of outcome following aSAH [14]. WFNS grade is not available in the UK Biobank but length of stay, which is strongly correlated with WFNS [15], was used as a surrogate.

### Mediation analysis

To explore whether significant fatigue mediated any component of the effect of aSAH on employment status, causal mediation analysis using a natural effects model was performed utilizing the package medflex [16]. This method has been shown to be superior when analysing a binary mediator and outcome [17]. A non‐parametric bootstrap procedure with 1000 samples was used to derive standard errors and *p* values. This was performed in the aSAH and matched control cohorts, additionally controlling for the Townsend deprivation score [18] (data field 189) and education status, dichotomized into people holding a college or university degree at the time of initial assessment in the UK Biobank or not (data field 6138). Employment status was dichotomized into good and poor, with poor employment defined as ‘unemployed’ or ‘unable to work because of sickness or disability’ (data field 6142). The proportion of the effect of aSAH status on employment mediated by fatigue was calculated using the method described by VanderWeele [19].

To provide context and assess the relative importance of fatigue to employment a further mediation analysis was performed exploring what proportion of the effect of aSAH status on employment was mediated by persistent headache, another common sequela of aSAH [20]. Headache was defined as present or absent using data field 6159 (‘In the last month have you experienced headache that interfered with usual activity?’) and the same causal mediation analysis was performed.

All analyses were performed in R (version 3.6.2, R Foundation for Statistical Computing).

## RESULTS

**FIGURE 1:**
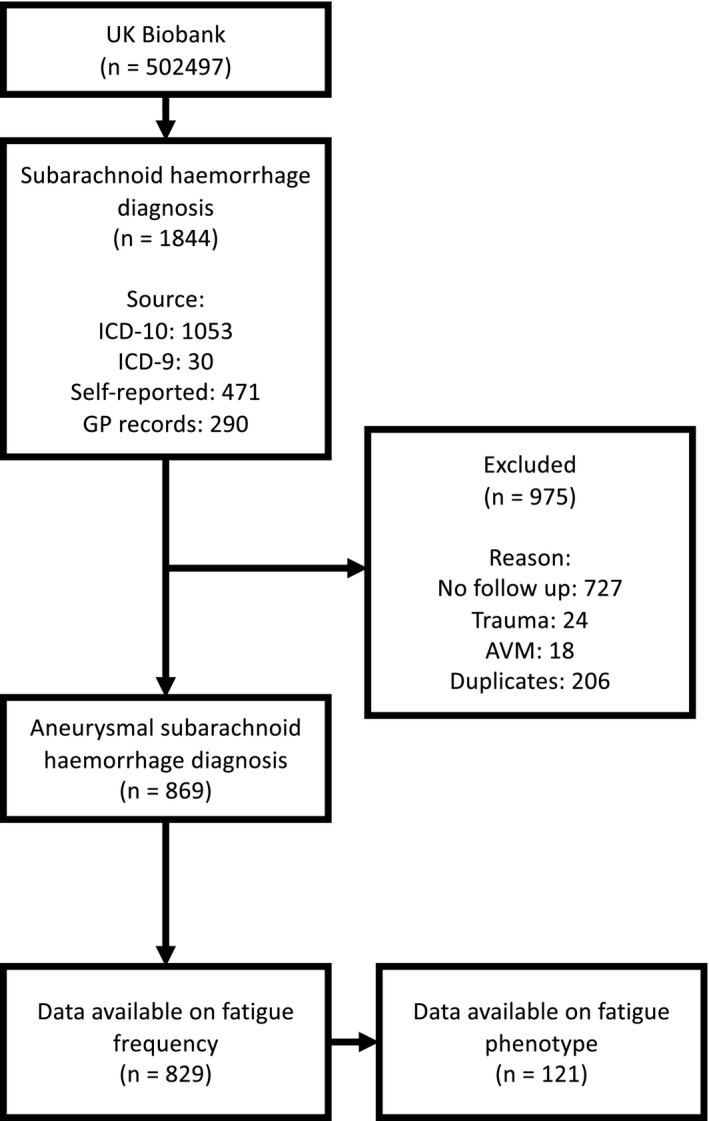
aSAH sample inclusion flowchart for UK Biobank

A total of 869 aSAH cases were identified from the UK Biobank. 829 were eligible for inclusion with data available on fatigue. 479,617 individuals were eligible for inclusion in the control cohort and 3316 controls were matched with a mean standard difference <0.004 (see Table 2 for demographics of individuals included in the study and Figure 1 for the flowchart of aSAH cases included).

**TABLE 2:** Demographics of aSAH and matched controls included in study

	aSAH cohort	Control cohort
Total sample size, *n*	829	3316
Subset completing phenotype questionnaire	121 (14.6%)	619 (18.7%)
Age at time of follow‐up		
Mean (±SD) years	58 (±7.1)	58 (±7.1)
Sex		
Male	336 (40.5%)	1348 (40.7%)
Female	493 (59.5%)	1968 (59.3%)
Depression or anxiety		
Present	326 (39.3%)	1308 (39.4%)
Absent	503 (60.7%)	2008 (60.1%)
Smoking status		
Current smoker	138 (16.6%)	556 (16.8%)
Not current smoker	691 (83.4%)	2760 (83.2%)
Education status		
College or university degree	223 (26.9%)	1032 (31.1%)
No college or university degree	605 (73.0%)	2262 (68.2%)
Missing	1 (0.0%)	22 (0.0%)
Townsend deprivation score		
Mean (±SD) months	−1.0 (±3.2)	−1.3 (±3.2)
Time to follow‐up		
Mean (±SD) months	123 (±116)	–
Length of stay		
Median (IQR) days	7 (11)	–
Missing	304 (36.7%)	–
Hydrocephalus	40 (4.8%)	–

Abbreviations: aSAH, aneurysmal subarachnoid haemorrhage; IQR, interquartile range; SD, standard deviation.

### Primary analysis

Significant fatigue was more frequent in cases compared to controls (aSAH 18.7%; controls 13.7%; *χ* ^2^ = 13.0, *p* < 0.001) at a mean follow‐up of 123 months. Length of stay and hydrocephalus were not significant predictors of fatigue following aSAH in this cohort (*p* = 0.940 and *p* = 0.150, respectively). After correction for multiple testing four fatigue phenotypes were more significant in the aSAH cohort compared to controls: ‘fatigue interferes with work, family or social life’, ‘fatigue is amongst the three most disabling symptoms’, ‘fatigue causes frequent problems’ and ‘easily fatigued’ (Table 3). This suggests that fatigue has an impact in almost all domains of life and significantly impairs a patient's quality of life.

**TABLE 3:** Comparison of fatigue phenotype questions between aneurysmal subarachnoid haemorrhage (aSAH) and control cohorts using the *t* test

Data field *Question*	Mean score in aSAH cohort	Mean score in control cohort	*p* value
120119 *Motivation is lower when fatigued*	5.07	4.94	0.50
120120 *Exercise brings on fatigue*	3.35	3.07	0.20
120121 *Easily fatigued*	3.88	3.36	0.014^a^
120122 *Fatigue interferes with physical functioning*	3.89	3.70	0.35
120123 *Fatigue causes frequent problems*	3.23	2.75	0.020^a^
120124 *Fatigue prevents sustained physical functioning*	3.36	2.96	0.059
120125 *Fatigue interferes with carrying out certain duties and responsibilities*	3.43	3.04	0.056
120126 *Fatigue is amongst the three most disabling symptoms*	3.42	2.81	0.0067^a^
120127 *Fatigue interferes with work, family or social life*	3.37	2.82	0.0089^a^

*Note*: Benjamini–Hochberg method with false discovery rate of 5% employed to correct for multiple testing.

^a^ Signifies significant *p* values.

**FIGURE 2:**
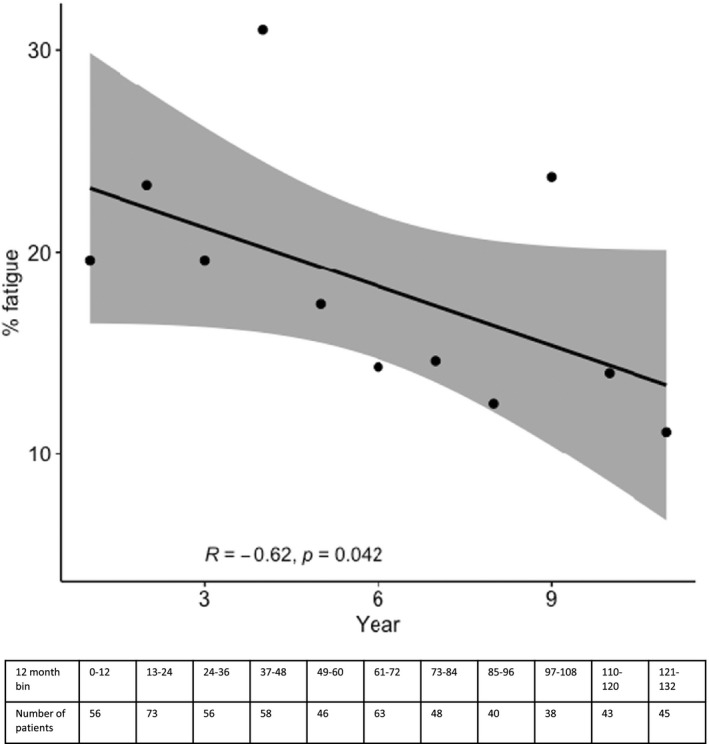
Change in frequency of fatigue over time, divided into 12‐month bins. Data beyond 11 years were not included due to the sparsity of data in each annual bin

The frequency of significant fatigue decreased by half from 19.6% in the first year following aSAH to 11.1% in the eleventh year with a significant relationship between frequency of fatigue and time (*R*_S_ = −0.62, *p* = 0.04, Figure 2).

### Mediation analysis

Unemployment or inability to work due to sickness/disability was significantly more frequent in the aSAH population (aSAH 18.7%; controls 5.9%; *χ* ^2^ = 138.9, *p* < 0.001). Mediation analysis identified that the estimated natural indirect effect of aSAH status on employment that was mediated by fatigue was significant, with an odds ratio of 1.21 (95% confidence interval [CI] 1.07–1.36, *p* = 0.001). The odds ratio for the estimated natural direct effect of aSAH status on employment was 2.97 (95% CI 2.51–3.49, *p* < 0.001). The proportion of the effect of aSAH on employment mediated by fatigue was 24.0%.

By comparison the estimated natural indirect effect of aSAH status on employment that was mediated by headache was significant, with an odds ratio of 1.06 (95% CI 1.02–1.11, *p* = 0.004). The proportion of the effect of aSAH on employment mediated by headache was 8.3%.

## DISCUSSION

In this large sample size, it is demonstrated that fatigue is more common following aSAH compared to matched controls and persists in the long term, with a mean follow‐up of over 10 years. Significant fatigue, defined as present for greater than 50% of the time, gradually improves over time in about half of patients, but has important implications. aSAH survivors report that it is one of the most disabling symptoms impacting quality of work, social and family life. In keeping with this, it is demonstrated that fatigue makes a large contribution to unemployment and inability to work due to sickness/disability following aSAH. This information will be helpful to counsel patients regarding the duration and prognosis of fatigue following aSAH and emphasizes the importance of management strategies to improve this disabling symptom and consequently promote a return to employment.

Kutlubaev et al. [7] reported a weighted mean frequency of fatigue of 73.6% in the first year following aSAH using five studies. This is much greater than the 19.6% reported in this study; however, a number of studies used by Kutlubaev et al. defined fatigue as present or absent based on a single binary question inflating the frequency of fatigue by including any self‐reported fatigue. In the present study fatigue is defined as significant if present for greater than 50% of the time and the frequency is in keeping with other studies that focus on the presence of significant fatigue [21, 22]. In the UK Biobank dataset, if fatigue is defined as the occurrence of any fatigue, it is present in 80.4% in the first year in keeping with the frequency reported by Kutlubaev et al. [7].

In this study it is shown that the frequency of fatigue significantly improves over time, decreasing by about 50% from around 20% in the first year to 10% after a decade. A recent study of 356 patients also reported that the prevalence of fatigue gradually decreased from 1 to 7 years post‐aSAH, although the decrease was not statistically significant [10]. In this study a larger sample size is included and a longer follow‐up explaining the greater significance of our results. It is also shown that length of stay, a surrogate of severity of clinical presentation, and hydrocephalus are not predictors of fatigue following aSAH. These results differ from the same recent study [10]. This may be because the UK Biobank favours good outcome individuals due to the requirement to engage in detailed follow‐up assessments. Both severity of clinical presentation and hydrocephalus are predictive of poor outcome [23] and are consequently underrepresented in this cohort, limiting our ability to study their association with fatigue. However, it may also be a real observation. It would be easy to rationalize that, once treated, hydrocephalus does not increase fatigue, supported by a further study which showed an association between acute but not chronic treatment of hydrocephalus [9]. Also, although it would be easy to assume more severe haemorrhages result in more severe fatigue, it is possible that patients with worse outcomes have lower activity levels and are more focused on their functional deficits and relatively underreport fatigue. This fits with our anecdotal observations that often some of the best performing patients are most limited by fatigue.

Unemployment is common following aSAH with up to 50% reporting impaired return to work [24]. A number of factors have been implicated in return to work following aSAH including independence at discharge, consciousness at admission [25] and cognitive deficits following aSAH [2]. Fatigue has also been implicated [8, 9] and this study emphasizes the importance of fatigue to employment by demonstrating that it mediates a significant proportion of the effect of aSAH on employment. The long follow‐up time (mean over 10 years) in this cohort further emphasizes the importance of fatigue as it has impact even at such a late stage after aSAH. To emphasize the importance of fatigue on employment the contribution of fatigue was compared to that of another common sequela of aSAH, persistent headache, demonstrating that fatigue is a much more dominant factor (24.0% vs. 8.3%). A previous study further supports the relative importance of fatigue with cognition also contributing a much smaller effect on employment (24.0% vs. 6.6% [2]).

Both fatigue and unemployment impair quality of life following aSAH [8, 26] emphasizing the importance of managing the symptom of fatigue following aSAH, especially as it persists in the long term and impacts employment. At present there are no pharmacological therapies to improve fatigue following stroke [27], but there are non‐pharmacological strategies which can improve the symptoms of fatigue [28]. Uptake of these strategies following aSAH in addition to ongoing pharmacological trials (e.g., NCT 03209830) may help to improve fatigue with subsequent benefits for survivors' employment and quality of life.

### Limitations

As UK Biobank participants are required to attend multiple very detailed assessment centre visits this study is biased towards individuals with a better outcome and more motivation. In comparison to poor outcome individuals who are preoccupied by functional deficits, aSAH cases included in this study are more likely to be aware of symptoms such as fatigue. Consequently, caution should be taken when applying these results to poorer outcome individuals.

In this study, a single question (‘Over the past 2 weeks, how often have you felt tired or had little energy?’) was used to assess frequency of fatigue. Future prospective studies should use more detailed assessments of fatigue, including validated tools such as the Chalder fatigue scale [29] or the fatigue severity scale [30], to provide greater insight into the nature of fatigue following aSAH. In addition, a number of factors have been shown to influence fatigue following aSAH including the presence of anxiety and depression [11]. In this study this is controlled for by matching cases and controls for the presence of anxiety/depression but more comprehensive fatigue assessment tools may be able to further elucidate the role of these factors in post‐aSAH fatigue. This study was also unable to assess change in fatigue on an individual level due to lack of serial measurement of fatigue and future studies should also include repeated measures of fatigue to give further detailed information on change in fatigue over time.

Finally, in this analysis, data were only available on employment status following aSAH and consequently it was not possible to study change in employment status before and after aSAH. This finding needs to be confirmed using employment data from individuals before and after aSAH.

## CONCLUSION

Fatigue is more common following aSAH compared to matched controls and persists in the long term. Fatigue gradually improves over time with significant fatigue decreasing by about 50% from around 20% in the first year to 10% after a decade. Fatigue negatively impacts quality of life and employment following aSAH. Further work is required to develop treatments and management strategies for fatigue following aSAH with a view to improving quality of life and employment.

## AUTHOR CONTRIBUTIONS

IG and DB conceived the study. All authors contributed to the study design. The first draft of the manuscript was written by BG and HC; all authors commented on previous versions of the manuscript. All authors read and approved the final manuscript.

## FUNDING INFORMATION

BG is funded by the Royal College of Surgeons of England, Society of British Neurological Surgeons, Barrow Foundation and Guarantors of Brain in addition to the Institute for Life Sciences, University of Southampton.

## CONFLICT OF INTEREST

None.

## Supporting information


Table S1

## Data Availability

The data that support the findings of this study are available from the UK Biobank (https://www.ukbiobank.ac.uk) by application.
