# Supplementary material for: Long‐term fatigue following aneurysmal subarachnoid haemorrhage and the impact on employment
Source: Eur J Neurol. 2022 Sep 12;29(12):3564–70. doi: 10.1111/ene.15533 (PMC9825863; doi:10.1111/ene.15533)
Supplement: Supplementary file 1 — Table S1 [file ENE-29-3564-s001.pdf]

Supplementary material

Long-term fatigue following aneurysmal subarachnoid haemorrhage and the impact on employment

Ben Gaastra*, Harry Carmichael, Ian Galea, Diederik Bulters
*corresponding author email: b.gaastra@soton.ac.uk

Data field	Code and definition
Inclusion	
Data field 41270<br>ICD 10 codes	I600 I60.0 Subarachnoid haemorrhage from carotid siphon and bifurcation<br><br>I601 I60.1 Subarachnoid haemorrhage from middle cerebral artery<br><br>I602 I60.2 Subarachnoid haemorrhage from anterior communicating artery<br><br>I603 I60.3 Subarachnoid haemorrhage from posterior communicating artery<br><br>I604 I60.4 Subarachnoid haemorrhage from basilar artery<br><br>I605 I60.5 Subarachnoid haemorrhage from vertebral artery<br><br>I606 I60.6 Subarachnoid haemorrhage from other intracranial arteries<br><br>I607 I60.7 Subarachnoid haemorrhage from intracranial artery, unspecified<br><br>I609 I60.9 Subarachnoid haemorrhage, unspecified<br>Ruptured aneurysm
Data field 41271<br>ICD9	430 Subarachnoid haemorrhage<br><br>4309 Subarachnoid haemorrhage
Data field 42040<br>Primary care data	G60 Equates to ICD-10 code I609<br><br>G600 Equates to ICD-10 code I607<br><br>G601 Equates to ICD-10 code I600<br><br>G602 Equates to ICD-10 code I601<br><br>G603 Equates to ICD-10 code I602<br><br>G605 Equates to ICD-10 code I604<br><br>G606 Equates to ICD-10 code I605<br><br>G60X Equates to ICD-10 code I607

	G60z Equates to ICD-10 code I609<br><br>Gyu60 Equates to ICD-10 code I606<br><br>Gyu6E Equates to ICD-10 code I607<br><br>X00Df Equates to ICD-9 code 430<br><br>X00Dg Equates to ICD-10 code I609<br><br>X204F Equates to ICD-10 code I609<br><br>Xa01c Equates to ICD-10 code I606<br><br>Xa01h Equates to ICD-10 code I601<br><br>Xa01i Equates to ICD-10 code I606<br><br>Xa01j Equates to ICD-10 code I602<br><br>Xa01k Equates to ICD-10 code I603<br><br>Xa01l Equates to ICD-10 code I604<br><br>Xa01m Equates to ICD-10 code I606<br><br>Xa01o Equates to ICD-9 code 430
Data field 20002<br>Self-reported medical conditions	1086 Subarachnoid haemorrhage
Exclusion	
Data field 41270<br>ICD 10 codes	Q282 Q28.2 Arteriovenous malformation of cerebral vessels<br><br>Q283 Q28.3 Other malformations of cerebral vessels<br><br>S-T Injury, poisoning and certain other consequences of external causes<br><br>V,W,X External causes of morbidity and mortality
Data field 41271<br>ICD9	74780 Arteriovenous aneurysm of brain<br><br>74781 Other anomalies of cerebral vessels<br><br>800-900 Trauma and injury
Data field 42040<br>Primary care data	P7y01 Equates to ICD-10 code Q282<br><br>P7y02 Equates to ICD-10 code Q283<br><br>S, U Equates to ICD-10 codes S,T,V,W,X and ICD-9 codes 800-900

Supplementary table 1. Inclusion and exclusion codes for aSAH cases in the UK Biobank. ICD: International Classification of Diseases.
